# Ochronosis

**Published:** 1909-07-24

**Authors:** 


### SPECIAL ARTICLE.

### OCHRONOSIS.

### A SHORT ACCOUNT OF A RARE FORM OF PIGMENTATION.

Abnormal pigmentation of the integuments, although often of obscure origin, is by no means an uncommon condition.

The black staining of cartilage to which the term Ochronosis has been applied a little inaptly, is, however, a condition so rare as to constitute little more than a pathological curiosity. Only about a dozen cases have been recorded, and even in the descriptions of these there is some diversity. Professor Osler, * in 1904 published a short account of three cases, two of his own, brother's, and one of Ogden's. Dr. Frank Pope, † of Leicester, also described a case which presented in addition the features of other allied conditions, and a note was appended to his article by Dr. Garrod, upon the association of Ochronosis with alkaptonuria. Here the matter assumes a practical aspect and becomes of distinct importance to the medical examiner for life assurance.

Ochronosis and Alkaptonuria.

One of Osler's cases was a man who had been rejected many times by the medical referees for insurance offices, and the other had been definitely diagnosed as "diabetes" by distinguished German and English physicians; yet, though the urine in each case held a copper-reducing substance, in neither was there a trace of dextrose.‡

Futcher, who investigated one of them, and has reported very fully, states that his attention was arrested by a curious deviation from the normal appearances in the performance of the Fehling test. He added a few drops of urine to some boiled Fehling's solution; it was immediately turned brownish-black, and when the reduction took place the precipitate was a lighter yellow than usual, whilst the supernatant fluid was not decolorised. He then found that the fermentation test, the phenyl-hydrazine, and the bismuth (Böttger's) test did not come off, nor was the urine optically active. Nevertheless, the Fehling reaction had led very competent observers, individually, to the erroneous diagnosis of glycosuria.

Dr. Pope points out that Ochronosis occurs in the degenerative period of life, whether that of true senility, or whether, as in many cases, that of precocious senility induced by wasting disease. Alkaptonuria is by no means always associated, and even were it so, it must not be forgotten that in itself it is not inimical to life.

Futcher§ quotes many cases to prove the accuracy of this contention; thus Brune had an example in a perfectly healthy young man of 29; Kirk observed the condition in three brothers of tender years; Baumann and Wolkow saw it in a male of 67 and a female of 60, in whom the passage of dark or darkening urine had been lifelong; Ebstein and Müller in a child aged eighteen months. Fürbringer's patient had miliary tuberculosis, and, most interesting of all, Geyger's patient had also glycosuria. This last observation recalls the "diabète bronzé" of Hanot, which is characterised by a pigmentation similar in some respects to the superficial coloration described in the cases of ochronosis. Opie and Osler, however, have described cases conforming to the type of diabète bronzé, without glycosuria; there is widespread hæmochromatosis, not only of skin but also of the liver, pancreas and spleen, together with enlargement and cirrhosis of these organs.

Robert Saundby some years ago described in the "Transactions of the Pathological Society of London" three cases occurring in a father, his son and daughter, who showed some excess of cutaneous pigmentation, and who all passed dark-coloured urine, which, in one instance at least, contained a copper-reducing substance. Two of them whom he examined had very considerable enlargement of the spleen, and in one patient, who died whilst under observation, there was found "atrophy" of the supra-renal.

Pigmentation and Addison's Disease.

The association of pigmentation with disease of the supra-renal is, of course, quite familiar under the name "Addison." Dr. Pope's case exhibited "tuberculosis of the lung and disease of adrenal glands (Addison's)," but the pigment, both in superficial distribution and in actual situation in the skin, was found unlike that of true Addison's disease. Instead of the deeper layers of the rete malpighii, it occupied the subjacent fibro-elastic bundles; a very similar position in fact to that held by the colouring matter of pigmented wens. There is no close relation between the pigment of Ochronosis, or the colouring substance in the urine, and melanin.

Dr. Garrod used the term alkaptonuria in the restricted sense of a urine containing homogentisic acid, or uroleucic acid, and not simply of one containing "a substance which reduces alkaline copper and greedily absorbs oxygen in the presence of an alkali turning black or brown," which was all that Bœdeker who introduced the term "alkaptone" intended to imply. Albrecht's case of ochronosis had urine which satisfied Bœdeker's definition but did not contain homogentisic acid, and we think with Guiart and Grimbert that the broader meaning might well be retained.

In Pope's case, though black urine was passed, this was probably due to the carbolic lotion with which for years the patient had dressed a chronic ulcer of the leg. Phenol and its derivatives not infrequently, although capriciously, determine the passage of dark urine, and this will sometimes reduce Fehling's solution.

* *Lancet*, January 2, 1904. † *Lancet*, January 6, 1906.
‡ *New York Med. Journ.*, January 15, 1898. § *Loc. Cit.*

